# Sexually dimorphic expression of Dmrt1 and γH2AX in germ stem cells during gonadal development in *Xenopus laevis*

**DOI:** 10.1002/2211-5463.12035

**Published:** 2016-02-26

**Authors:** Kazuko Fujitani, Asako Otomo, Mikako Wada, Nobuhiko Takamatsu, Michihiko Ito

**Affiliations:** ^1^ Department of BioscienceSchool of ScienceKitasato UniversitySagamiharaJapan; ^2^ Department of Molecular Life SciencesTokai University School of MedicineIseharaJapan

**Keywords:** germ cells, oogonia, sexual dimorphism, spermatogonia, transcription factor, *Xenopus laevis*

## Abstract

In many animals, primordial germ cells (PGCs) migrate into developing gonads. There, they proliferate and differentiate into female and male germ stem cells (GSCs), oogonia and spermatogonia, respectively. Few studies have focused on the molecular mechanisms underlying the development of GSC sex determination. Here, we investigated the expression of the transcription factor Dmrt1 and a phosphorylated form of the histone variant H2AX (γH2AX) during gonadal development in *Xenopus laevis*. During early sexual differentiation, Dmrt1 was expressed in the GSCs of the ZW (female) and ZZ (male) gonads as well as somatic cells of the ZZ gonads. Notably, the PGCs and primary GSCs contained large, unstructured nuclei, whereas condensed, rounder nuclei appeared only in primary oogonia during tadpole development. After metamorphosis, Dmrt1 showed its expression in secondary spermatogonia, but not in secondary oogonia. Like Dmrt1, γH2AX was expressed in the nuclei of primary GSCs in early developing gonads. However, after metamorphosis, γH2AX expression continued in primary and secondary spermatogonia, but was barely detected in the condensed nuclei of primary oogonia. Taken together, these observations indicate that spermatogonia tend to retain PGC characteristics, compared to oogonia, which undergo substantial changes during gonadal differentiation in *X. laevis*. Our findings suggest that Dmrt1 and γH2AX may contribute to the maintenance of stem cell identity by controlling gene expression and epigenetic changes, respectively.

**Abbreviations**
BrdU: 5‐bromo‐2′‐deoxyuridine
DSB: double‐strand break
GSC: germ stem cell
PGC: primordial germ cells
RT‐qPCR: quantitative RT‐PCR

Sexual dimorphism normally occurs after gonadal sex determination in vertebrates. Gonadal sex is determined by sex‐determining genes on the sex chromosome, such as mammalian Y‐linked *Sry*, chicken Z‐linked *Dmrt1*, the African clawed frog W‐linked *Dm‐W*, and teleost fish medaka Y‐linked *dmy/dmrt1by* 1, 2, 3, 4. It is generally believed that these sex‐determining genes are expressed in the somatic cells of gonads, leading to primary ovarian or testicular formation. After sex determination, primordial germ cells (PGCs) differentiate into oogonia or spermatogonia (female and male germ stem cells [GSCs]). However, the molecular and morphological differences between the female and male GSCs in many vertebrates remain unclear.

*Dmrt1* encodes a transcription factor characterized by the presence of a DNA‐binding domain called the DM domain. Dmrt1 induces testis formation, and is conserved across vertebrate species 4, 5, 6, 7, 8. Its paralogs include the *X. laevis* and medaka sex‐determining genes, *dm‐W* and *dmy*/*dmrt1bY*. In mice, Dmrt1‐deficient Sertoli cells are reprogrammed into granulosa cells during postnatal development, indicating that Dmrt1 plays an important role in the regulation of somatic cell masculinization 9. Thus, Dmrt1 might be a masculinizing master gene in vertebrates. Dmrt1 also contributes to the development of both female and male germ cells in mice. Curiously, mouse Dmrt1 negatively controls meiosis in male germ cells by repressing *Stra8* transcription 10, but promotes meiosis in female germ cells by enhancing *Stra8* transcription 11. However, there have been few reports investigating Dmrt1's functions in the GSCs of other vertebrate species.

Another factor involved in germ cell development is γH2AX, a phosphorylated form of the H2A variant, H2AX. γH2AX is induced by double‐strand breaks (DSBs) in DNA, and contributes to DNA repair in mitotic cells. In several organisms, γH2AX is also involved in meiotic recombination and/or sex chromosome inactivation from the leptotene to diplotene stages in meiotic germ cells 12, 13. To date, the presence of γH2AX has been demonstrated in spermatogonia 14, but not in PGCs or oogonia. In addition, the function of γH2AX has not been understood in these GSCs yet.

To investigate the role of these two factors in the development of sexual dimorphism in GSCs and somatic cells in *X. laevis*, we performed immunostaining with antibodies against Dmrt1 and γH2AX using genetically female (ZW) and male (ZZ) gonads from various stages of development. During the early stages of sex differentiation, Dmrt1 and γH2AX were expressed in both the ZW and ZZ GSCs. In vitellogenic ovaries, the nuclei and cell bodies of the female GSCs underwent condensation, and γH2AX expression was barely detected in the condensed nuclei after metamorphosis, but was still detected in the nuclei of male GSCs. Similarly, Dmrt1 expression in male GSCs continued during development, but was confined to only certain types of female GSCs. Finally, we discuss sexual dimorphism between female and male GSCs, and the potential functions of Dmrt1 and γH2AX in the GSCs during develoment.

## Results

### *dmrt1* is expressed at similar levels in ZZ and ZW gonads during early sex differentiation

**Figure 1:**
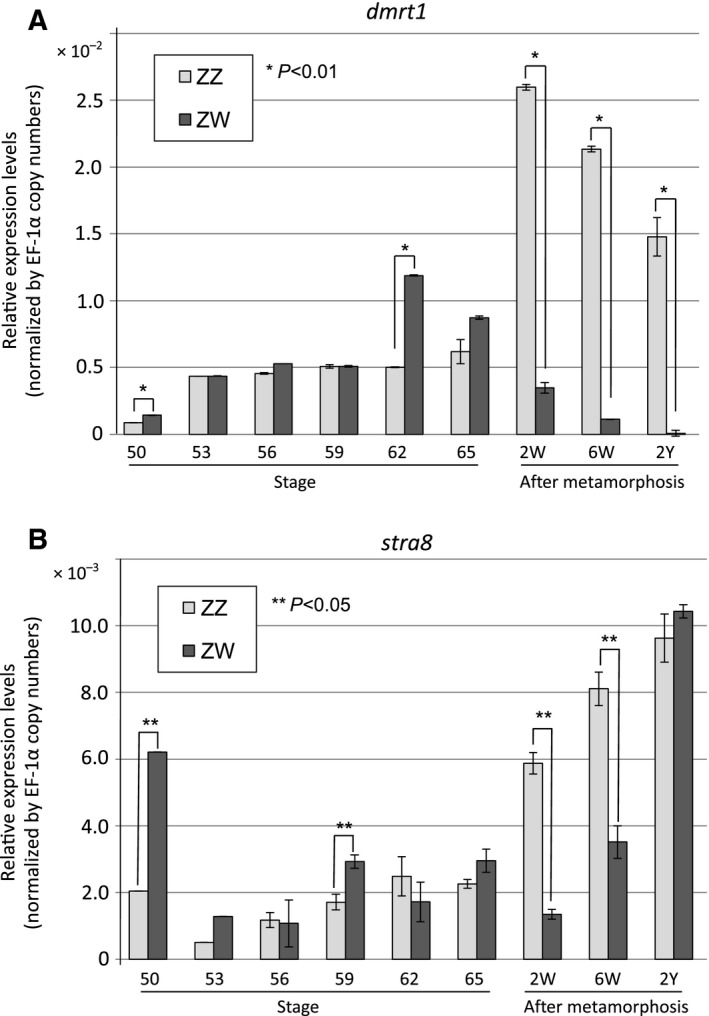
Real‐time PCR analysis of *dmrt1* and *stra8* in ZZ and ZW gonads during sexual development. qPCR was performed for *dmrt1* (A) or *stra8* (B) using cDNAs derived from the total RNAs of three ZZ or ZW gonads from tadpoles at different stages of development and adult frogs. The *dmrt1* or *stra8* primer pairs were designed within common sequences shared between the two *dmrt1* (*dmrt1*.*L* and *dmrt1*.*S*) and two *stra8* cDNAs in *X. laevis*. W, weeks; Y, years.

To examine the *dmrt1* expression during ZZ and ZW gonadal development after sex determination, we conducted quantitative RT‐PCR (RT‐qPCR) experiments using total RNA samples from pools of ZW and ZZ gonads at various developmental stages. At stage 50, just after sex determination, *dmrt1* expression was slightly higher in the ZW gonads than in the ZZ gonads. However, during most stages of tadpole development, it was expressed at similar levels in the ZW and ZZ gonads (Fig. 1). After metamorphosis, *dmrt1* expression was increased in the ZZ gonads, and decreased in the ZW gonads. These results suggest that Dmrt1 may play a role in early gonadal development in both sexes. We also examined the *stra8* expression by RT‐qPCR using the same RNA samples, because *Stra8* is a target of Dmrt1 in female and male premeiotic germ cells in mice 10, 11. A similar expression pattern was observed for the two genes, except for the cases at stage 50 or 2 years.

### Dmrt1 is expressed in both female and male GSCs, but only in male somatic cells, during tadpole development

**Figure 2:**
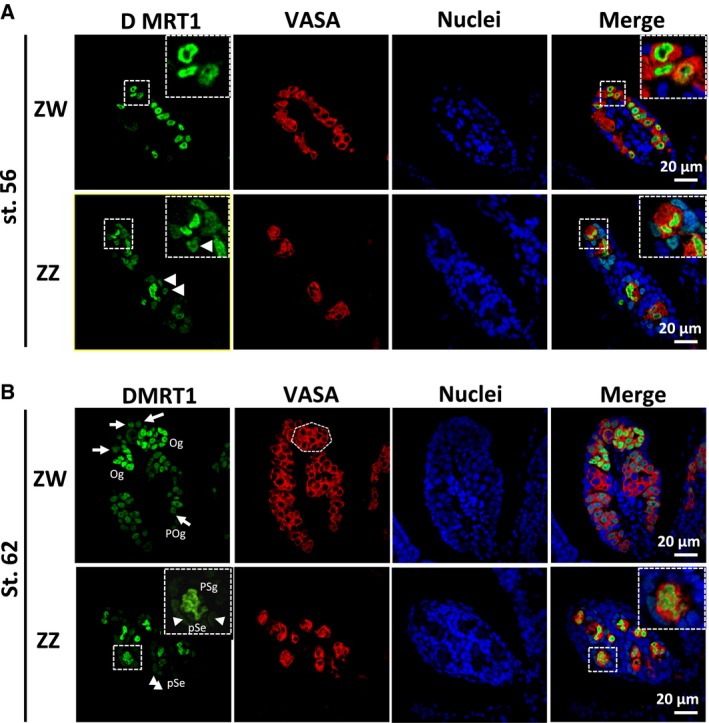
Distribution of Dmrt1 in ZZ and ZW gonads during tadpole development. Immunostaining with anti‐Dmrt1 and anti‐VASA antibodies was performed on gonadal sections from ZW and ZZ tadpoles at stages 56 (A) and 62 (B). Nuclei were stained with TO‐PRO‐3. Oc, oocyte; Og, secondary oogonium; POg, primary oogonium; PSg, primary spermatogonium; pSe, pre‐Sertoli cell. White arrowheads and arrows indicate Dmrt1‐expressing pre‐Sertoli cells and primary oogonia, respectively. Typical GSCs with somatic cells and their 2.5× magnification images are shown in the dashed squares. A dashed polygon indicates a typical cyst consisting of secondary oogonia.

During the early stages of gonadal differentiation, after sex determination (stages 52–58), both GSCs and somatic cells actively proliferate in the ZZ and ZW gonads, which share a similar morphology. Female GSCs are located in the cortical region of the ZW gonads, while male GSCs are mainly located in the medulla in the ZZ gonads 3. During these stages, the nuclei of the ZZ and ZW GSCs were large and unstructured, similar to their morphologies in the PGCs of the female and male gonads before sex determination (Fig. 2A). To clarify the localization of Dmrt1 in the developing gonads, we performed immunohistochemical analysis using stage‐56 tadpoles and an anti‐Dmrt1 antibody, as well as an antibody to VASA, a germ‐line‐specific protein. Dmrt1 was highly expressed in the large, unstructured nuclei (green) in both the primary oogonia and spermatogonia, while VASA was expressed in the cytoplasm (red) of these cells (Fig. 2A). The strong expression of Dmrt1 in both the ZZ and ZW GSCs was consistent with the results of our quantitative RT‐PCR experiments (Fig. 1). Importantly, weaker, but substantial Dmrt1 expression was observed in the somatic cells surrounding the primary spermatogonia in the ZZ gonads at stage 56 (Fig. 2A, arrows). In contrast, there was no detectable Dmrt1 expression in the somatic cells of the ZW gonad. These findings supported a role for Dmrt1 in GSC development and a male‐specific function in somatic cell masculinization, which leads to testicular development after sex determination 5.

### Primary oogonia exhibit nuclear and cell body morphological changes during tadpole development

During the later stages of metamorphosis (stages 59–65), differences between male and female germ cell morphologies, but not tissue morphologies were observed. At stage 62, many cysts consisting of secondary oogonia, which were proliferating or entering their first meiosis, were observed in the ZW gonads by staining for Dmrt1 and VASA (Fig. 2B). Notably, the primary oogonia underwent significant changes in cell morphology: their cell bodies shrank and their large, distorted nuclei became more condensed, taking on a round or elliptical shape (Fig. 2A). However, they were still located in the cortex of the gonads. Dmrt1 was expressed in the primary oogonia (arrows in Fig. 2B) and in subpopulations of secondary oogonia in the cysts, but was barely detected in the primary oocytes (Fig. 2).

In the ZZ gonads, the primary structure of the testis cord was evident at these stages. The primary spermatogonia were surrounded by slender somatic cells (pre‐Sertoli cells). As expected, Dmrt1 was highly expressed in the primary spermatogonia, whereas its expression in the surrounding pre‐Sertoli cells (arrowheads in Fig. 2B) was relatively low. The nuclei of the primary spermatogonia remained large and distorted (Fig. 2B).

### Meiotic female and male germ cells express little or no Dmrt1 after metamorphosis

**Figure 3:**
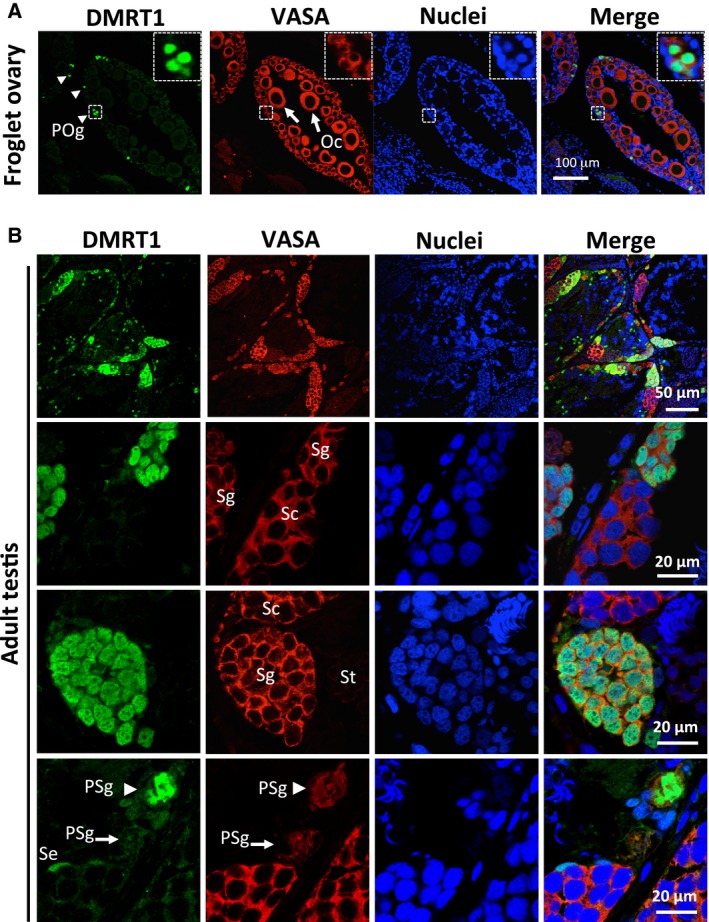
Distribution of Dmrt1 in the immature ovary and mature testis after metamorphosis. Immunostaining with anti‐Dmrt1 and anti‐VASA antibodies was performed using the immature ovary from a ZW frog 2 weeks after metamorphosis (A) and the mature testis of an adult ZZ frog (B). Nuclei were stained with TO‐PRO‐3. Oc, Oocyte; POg, primary oogonium; PSg, primary spermatogonium; Se, Sertoli cell; Sg, secondary spermatogonium; Sc, spermatocyte; St, spermatid. pSe, pre‐Sertoli cell. Dmrt1‐expressing primary oogonia and their 5× magnification images are shown in the dashed squares. White arrows and arrowhead in (A) indicate primary oogonia and diplotene oocytes, respectively. White arrowheads in (B) indicate Dmrt1‐expressing primary spermatogonium.

After metamorphosis, the female and male gonads (ovaries and testes) show distinct differences in gross morphology. In the ZW ovaries, a sequence of hollow segmental cords, called ovarial sacs is formed. At the microscopic level, diplotene oocytes during vitellogenesis are frequently observed in the region surrounding the ovarian cavities. However, the oogonia remain in the cortex. Immunohistochemical analysis of ovarian Dmrt1 expression 2 weeks after metamorphosis revealed that Dmrt1 was expressed in the primary oogonia with condensed nuclei (Fig. 3A). In contrast, there was no detectable Dmrt1 expression in the somatic cells or oocytes at various stages including diplotene (Fig. 3A).

Analysis of the ZZ testes showed that their cephalocaudal dimensions were shorter after metamorphosis. Several types of cysts appeared during germ cell development, which consisted of synchronized secondary spermatogonia, spermatocytes, or spermatids. Dmrt1 was expressed in the primary spermatogina, secondary spermatogonia, and Sertoli cells (Fig. 3B), but not in the spermatocytes or spermatids in the immature testes 1 month after metamorphosis (data not shown) or in these cells in mature testes (Fig. 3B). Notably, the primary spermatogonia consisted of Dmrt1‐high and ‐faint expressing cells (Fig. 3B, arrowhead and arrow, respectively).

### Dmrt1 expression is not directly involved in GSC proliferation

**Figure 4:**
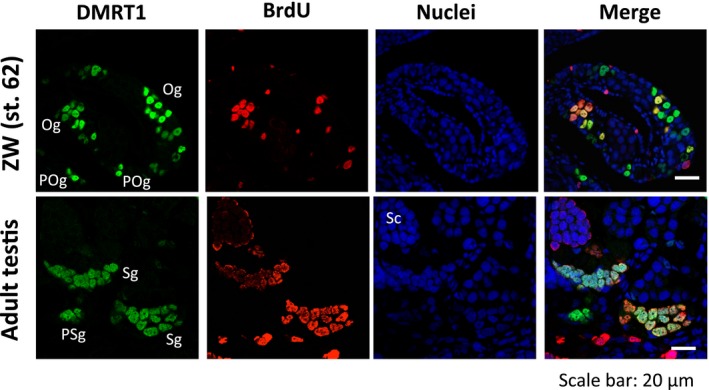
BrdU incorporation and Dmrt1 expression in the ZW developing ovary and ZZ adult testis. Immunostaining with anti‐Dmrt1 and anti‐BrdU antibodies was performed on the immature tadpole ovary at stage 62 and the mature testis of an adult frog. Nuclei were stained with TO‐PRO‐3. Og, secondary oogonium; POg, primary oogonium; PSg, primary spermatogonium; Sg, secondary spermatogonium; Sc, spermatocyte.

Since Dmrt1 was expressed in some cysts consisting of synchronized secondary oogonia in immature ovaries and in some primary spermatogonia in the mature testis (Fig. 3), we sought to determine if there was a relationship between Dmrt1 expression and cell proliferation. We labeled proliferating cells in the immature and mature gonads by injecting BrdU into stage‐62 tadpoles and adult frogs, and detecting the labeled cells with an anti‐BrdU antibody. In the immature ovary, some, but not all of the Dmrt1‐expressing secondary oogonia were BrdU‐positive, and many Dmrt1‐expressing primary oogonia were BrdU‐negative (Fig. 4). In the adult testis, Dmrt1 expression in the secondary spermatogonia was not strongly related to the degree of BrdU incorporation (Fig. 4). In addition, there was no relationship between Dmrt1 expression and BrdU incorporation in the primary spermatogonia.

### A phosphorylated form of H2AX (γH2AX) and Dmrt1 are coexpressed in primary spermatogonia and oogonia

**Figure 5:**
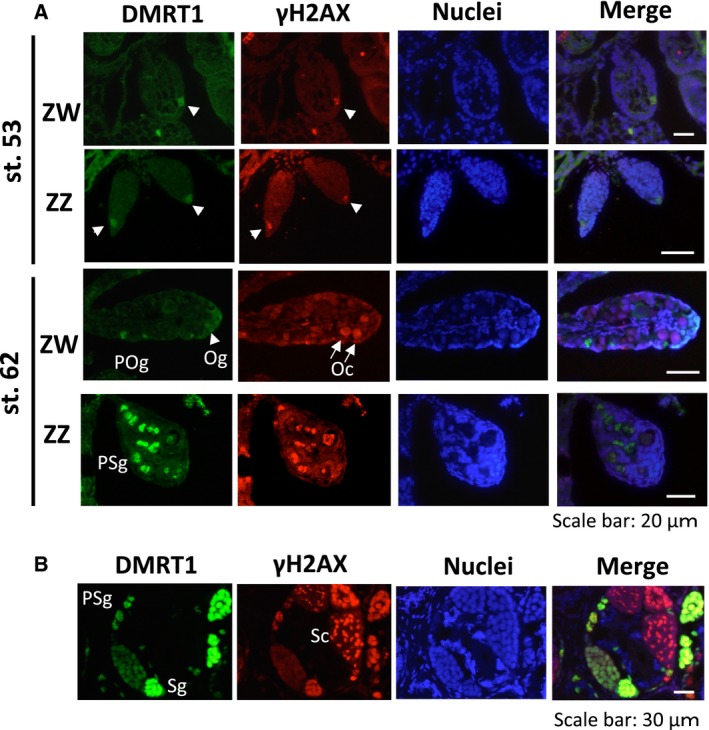
Distribution of γH2AX in ZZ and ZW gonads during development. Immunostaining with anti‐Dmrt1 and anti‐γH2AX antibodies was performed on gonadal sections from ZW and ZZ tadpoles at stages 53 and 62 (A) and on gonadal sections from a ZZ adult frog (B). Nuclei were stained with TO‐PRO. Oc, oocyte; POg, primary oogonium; PSg, primary spermatogonium; Sg, secondary spermatogonium; Sc, spermatocyte. White arrowheads and arrows indicate Dmrt1‐ or γH2AX‐positive primary GSCs and leptotene/zygotene oocytes, respectively.

γH2AX, which is a phosphorylated form of the histone variant H2AX, is involved in DNA repair in most cells or meiosis in both female and male germ cells. γH2AX is also expressed in embryonic stem cells and neural stem cells in mammals 15, 16. To examine the γH2AX expression in GSCs, we performed an immunohistochemical analysis of ZZ and ZW gonads at various developmental stages using both anti‐γH2AX and anti‐Dmrt1 antibodies. γH2AX was markedly expressed in the primary spermatogonia and oogonia, but not in the somatic cells of the ZZ and ZW gonads at stage 53, just after sex determination (Fig. 5A). The colocalization of γH2AX and Dmrt1 was observed in the nuclei of the primary GSC cells.

### γH2AX is differentially expressed in female and male GCSs after metamorphosis

In the ZW gonads at stage 62, the γH2AX signal was markedly expressed in the primary oocytes (Fig. 5A). The same pattern of γH2AX expression was observed at later stages of gonadal development. In contrast, γH2AX was expressed at similar levels in the primary spermatogonia at stages 53 and 62. In the adult testis, γH2AX was expressed in some cysts consisting of synchronized spermatocytes or secondary spermatogonia, as well as in the primary spermatogonia (Fig. 5B). Taken together, our findings suggested that DMRT1 and γH2AX are coexpressed in primary and secondary spermatogonia throughout testicular development and that γH2AX is differentially expressed in female and male GSCs after metamorphosis.

## Discussion

The transcription factor Dmrt1 has the potential to both activate and repress the expression of its target genes. In the somatic cell masculinization in mouse postnatal gonads, Dmrt1 activates the expression of *Sox8*,* Sox9*, and *Ptgdr*, and represses the transcription of *Foxl2*,* Esr1/2*,* Wnt4*, and *Rspo1* 9. In mouse germ cells, it positively and negatively regulates the expression of *Stra8*, which is involved in the premeiotic phases of oogenesis and spermatogenesis, respectively 10, 11. It has also been reported that Dmrt1 protects male GSCs against pluripotency and apoptosis in mice 17. In *X. laevis*, Dmrt1 was expressed in the primary oogonia and spermatogonia at an early stage of sex differentiation (Fig. 2), but its expression was reduced or absent in some subpopulations of secondary oogonia (Figs 2 and 3). Moreover, Dmrt1 expression was not involved in the proliferation of primary or secondary GSCs in either sex (Fig. 4). Collectively, these findings suggest that *X. laevis* Dmrt1 may be involved in maintaining GSC identity in male primary spermatogonia throughout development, and in female primary oogonia before metamorphosis, possibly by negatively or positively regulating apoptosis‐ and pluripotency‐promoting genes or GSC‐maintaining genes, respectively. Dmrt1 might also prevent entry into premeiotic S‐phase in secondary spermatogonia by regulating the *stra8* expression in *X. laevis*, as it does in mice. The absence of Dmrt1 from some secondary oogonia in *X. laevis* indicates that it does not function as a meiotic regulator in these cells. The real‐time PCR analysis revealed that the *dmrt1* and *stra8* mRNAs shared similar expression patterns during gonadal development (Fig. 1). Interestingly, *stra8* was expressed in the ZW and ZZ gonads at stages 50 and 53, which have no premeiotic germ cells. Although it is assumed that Stra8 function is limited to meiosis in germ cells in mammals, Stra8 might play another role in gonadal development in *X. laevis*.

The phosphorylation of H2AX, which generates γH2AX, was originally identified as an early event after ionizing radiation‐induced DNA DSBs 18. Meiotic DNA DSBs may also induce the generation of γH2AX. Here, we observed γH2AX expression not only in meiotic germ cells but also in female and male primary GSCs during early sex differentiation (Fig. 5). γH2AX was recently reported to be expressed in mammalian embryonic stem cells (ESCs), induced pluripotent stem cells, and neural stem cells 15, 16, 19. Turinetto and Giachino 20 suggested that γH2AX may contribute to the creation of specific chromatin structures in response to other cellular signals besides DNA damage. Thus, our results, in combination with previous findings, suggest that γH2AX may play an important role in maintaining stem cell identify by regulating epigenetic changes in various types of stem cells.

Very recently, Nishimura *et al*. 21 reported that *foxl3* shows specific expression in female GSCs throughout development, which would determine female germ cell identity for oocytes in the teleost fish medaka. Our experiments revealed substantial differences in morphology and protein expression between female and male GSCs during gonadal development in *X. laevis*. We found that γH2AX was barely detectable in female GSCs after metamorphosis. We also observed that primary oogonia become more condensed as ovarian development progressed, whereas primary spermatogonia exhibited a similar morphology to the PGCs throughout testicular development (Fig. 2). The tendency of male GSCs to maintain the PGC phenotype, compared with the phenotypic changes exhibited by female GSCs, could involve epigenetic differences and/or differences in the ovarian and testicular environments. It will be interesting to examine whether these sexual dimorphisms are common in other vertebrate species. If these differences are conserved, they may represent a mechanism for reducing ‘male‐driven evolution’, which refers to the higher number of cell divisions in the male germ line compared to the female germ line, and the higher prevalence of male germ‐line mutations 22, 23. In mice, correlation between p53 and Dmrt1 is involved in cell fate and identity of male GSCs 17. Accordingly, there might be stronger relationships between the DNA repair system and germ cell identity mediated through p53‐γH2AX and Dmrt1 in males than in females in *X. laevis*. It will be intriguing to study how the relationships could protect germ cells from mutations or whether they are conserved in vertebrates.

## Materials and methods

### Animal care and use

All of the experimental procedures involving *X. laevis* were approved by the Institutional Animal Care and Use Committee of Kitasato University. *X. laevis* frogs at various developmental stages were purchased from Watanabe Zoushoku (Yachiomachi, Japan) and maintained at 22 °C. Tadpole developmental stages were identified according to the descriptions by Nieuwkoop and Faber 24.

### Tissue sample preparation

Gonadal tissues were isolated from *X. laevis* tadpoles and adults at different stages of development. Tissue samples were fixed in 4% paraformaldehyde solution (4% paraformaldehyde, 70 mm phosphate buffer [pH 7.3]) and embedded in paraffin. Paraffin sections (7 μm) were generated and used for immunohistochemical experiments.

### Real‐time reverse transcription PCR (RT‐PCR)

Total RNAs were isolated from the tissues of ZZ and ZW gonads at various stages of development, using the RNeasy Mini Kit (Qiagen, Venlo, Netherlands). RNA (1 μg) was reverse transcribed with the Transcriptor First Strand cDNA Synthesis Kit (Roche, Basel, Switzerland), according to the manufacturer's instructions. Real‐time PCR was carried out using the SYBR Green Realtime PCR Master Mix (ToYoBo, Osaka, Japan). The *dmrt1* or *stra8* cDNA was amplified using the following primer pair: 5′‐GGGATTGCCAGTGCAAAAAG‐3′ (forward) and 5′‐TTCCAGCATCAAGCAAGAGC‐3′ (reverse) or 5′‐TACCTCAGCCAGGAGTGTG‐3′ (forward) and 5′‐TGTCCATAGTCTGCTGGTAG ‐3′ (reverse), respectively.

### Determining ZW or ZZ Status in *X. laevis*

The ZZ or ZW status of the gonads was determined by genomic PCR as described previously in Discussion.

### Antibodies

The mouse monoclonal anti‐VASA antibody and rabbit polyclonal anti‐Dmrt1 antibody were produced using the *X. laevis* VASA‐like protein and Dmrt1 as antigens, and were described elsewhere 5, 25. The anti‐phospho‐Histone H2A.X (Ser139) and anti‐BrdU (5‐bromo‐2′‐deoxyuridine) rabbit polyclonal antibodies were purchased from EMD Millipore (Billerica, MA, USA) and Cell Signaling Technology (Beverly, MA, USA), respectively. Alexa 488‐ and 594‐conjugated goat anti‐mouse and anti‐rabbit IgG antibodies were from Invitrogen (Carlsbad, CA, USA).

### Immunohistochemistry

The paraffin sections were deparaffinized in xylene followed by rehydration in a graded ethanol series. After washing in H_2_O, the sections were boiled in 10 mm citrate buffer (pH 6.0) for antigen unmasking. The sections were then incubated overnight at 4 °C with anti‐Dmrt1 [1 : 1000] and anti‐VASA [1 : 500] antibodies, which were diluted in PBS (without calcium or magnesium) containing 0.2% skim milk and 0.05% Triton X‐100. Alexa 488‐ and 594‐conjugated goat anti‐[mouse IgG (1 : 2000)] and anti‐[rabbit IgG (1 : 2000)] antibodies (Invitrogen) were used to detect the primary antibodies. Images of 0.2‐μm optical sections were captured and analyzed by the (Carl Zeiss Microscopy, Goettingen, Germany).

### BrdU incorporation

Tadpoles or frogs were injected intraperitoneally with BrdU (diluted in 70 mm phosphate buffer) at 600 μg·g^−1^ body weight, and sacrificed 4 or 24 h later. Tissue sections were deparaffinized and incubated in 1 m NaCl at 37 °C for 2 h, followed by neutralization in 0.1 m borate buffer (pH 8.5). The sections were blocked and then incubated overnight at 4 °C with anti‐BrdU (1 : 200) and anti‐Dmrt1 [1 : 10 000] antibodies. The sections were washed, and then the signals were detected as described above.

### Statistical analysis

All values are expressed as the mean ± SEM. Statistical analysis was performed using the Student's *t* test. *P* values < 0.01 were considered statistically significant.

## Author contributions

K.F., A.O, and M.I. designed research; K.F., M.W. and A.O performed research; K.F., A.O, N.T, and M.I analyzed data; and K.F., A.O, and M.I. wrote the paper.
